# Practical applications of ultrasound B-scan in vitreoretinal conditions: A narrative review

**DOI:** 10.1177/11206721261452073

**Published:** 2026-05-19

**Authors:** Antony Raharja, James Gooch, Asterios Diafas, Hadi Zambarakji, Ben Kirkpatrick

**Affiliations:** 1 4960 Moorfields Eye Hospital, London, UK; 2 156708 Whipps Cross University Hospital, Barts Health NHS Foundation Trust, London, UK; 3 59899 UCL Institute of Ophthalmology, London, UK

**Keywords:** Ultrasonography, ultrasound biomicroscopy, cataract surgery, dislocated intraocular lens, vitreous haemorrhage, retinal detachment, choroidal effusion, suprachoroidal haemorrhage.

## Abstract

Ocular ultrasonography is a key imaging modality when the posterior segment cannot be directly visualised by ophthalmoscopy. Image quality and interpretation are operator-dependent. Thus, good technique and understanding of the strengths and limitations of the different ultrasonography modalities e.g. A-scan (time-amplitude), B-scan (brightness), doppler and ultrasound biomicroscopy are valuable, especially in complex cases with diagnostic uncertainty. This article discusses the practical applications of ultrasonography in the diagnosis and management of common vitreoretinal emergencies (vitreous haemorrhage, retinal detachment and endophthalmitis) and complications of anterior segment surgery (choroidal effusion, suprachoroidal haemorrhage, malpositioned or dislocated lens).

## Introduction

Ultrasonography (USG) is a key imaging modality when the posterior segment cannot be directly visualised with ophthalmoscopy. Its value lies in the ability of sound waves to penetrate media opacities such as corneal oedema, hyphaema, pupillary membrane, mature cataract, and vitreous abnormalities (vitritis and haemorrhage). This enables real-time dynamic assessment for diagnosis and provides records for monitoring and medicolegal purposes.

The role of USG in ocular oncology and trauma has been extensively described elsewhere.^1–3^ Hence, this narrative review mainly discusses the practical applications of ultrasound in common vitreoretinal (VR) emergencies and complications of anterior segment surgeries which may require management by vitreoretinal surgeons. We specifically focus on B-scan (brightness) and its role and limitations under these conditions.

## Basic principles of ocular ultrasonography

Ultrasonography relies on differences in the absorption (attenuation) and reflectance of sound waves by intraocular structures and pathologies. A-scan (time-amplitude) is commonly used to measure the axial length for biometric intraocular lens calculation. The ability to measure thickness (distance between two points) and characterise internal echoes within ocular lesions is particularly valuable in ocular oncology. In VR conditions, A-scan can help to differentiate posterior vitreous detachment (PVD) from retinal detachment (RD), as the posterior hyaloid membrane shows lower reflectivity than the retina which demonstrates 90–100% reflectivity. However, the role of A-scan alone in VR emergencies may be limited without B-scan images to confirm probe alignment.

B-scan provides a two-dimensional cross-sectional grayscale image. The image quality is both setting- and operator-dependent. Simple adjustments of depth and gain are crucial to optimise image quality. Selecting depth penetration at least to the level of the retina or area of interest is essential because inadequate penetration results in poor image quality. The gain refers to the amplification of incoming (reflected) ultrasound signals detected by the receiver. Increasing the gain (measured in decibels, dB) makes the image brighter, enabling detection of less reflective structures such as mild vitreous opacities at the expense of reduced contrast resolution and increased artefacts, i.e. ‘noise’. It is sometimes helpful to start with higher gain to uncover subtle pathologies within the vitreous or vitreoretinal interface before decreasing gain to examine the retina. Other adjustable settings include power level (which changes the output from transducer) and frame averaging (which reduces noise but may also reduce the visibility of vitreous opacities in the image).

The area examined by B-scan is dependent on the placement and orientation of the ultrasound probe, as determined by the marker (Figure 1). By examining the eye in different orientations (axial, transverse, and longitudinal), the operator can build a three-dimensional mental image of the intraocular structure of interest. The clinical history and, if available, slit-lamp examination findings may direct the ultrasonography by identifying the area to be evaluated. However, in the absence of fundal view, it is important to take a systematic approach when performing a B-scan examination so as to not miss any pathology.

**Figure 1.**
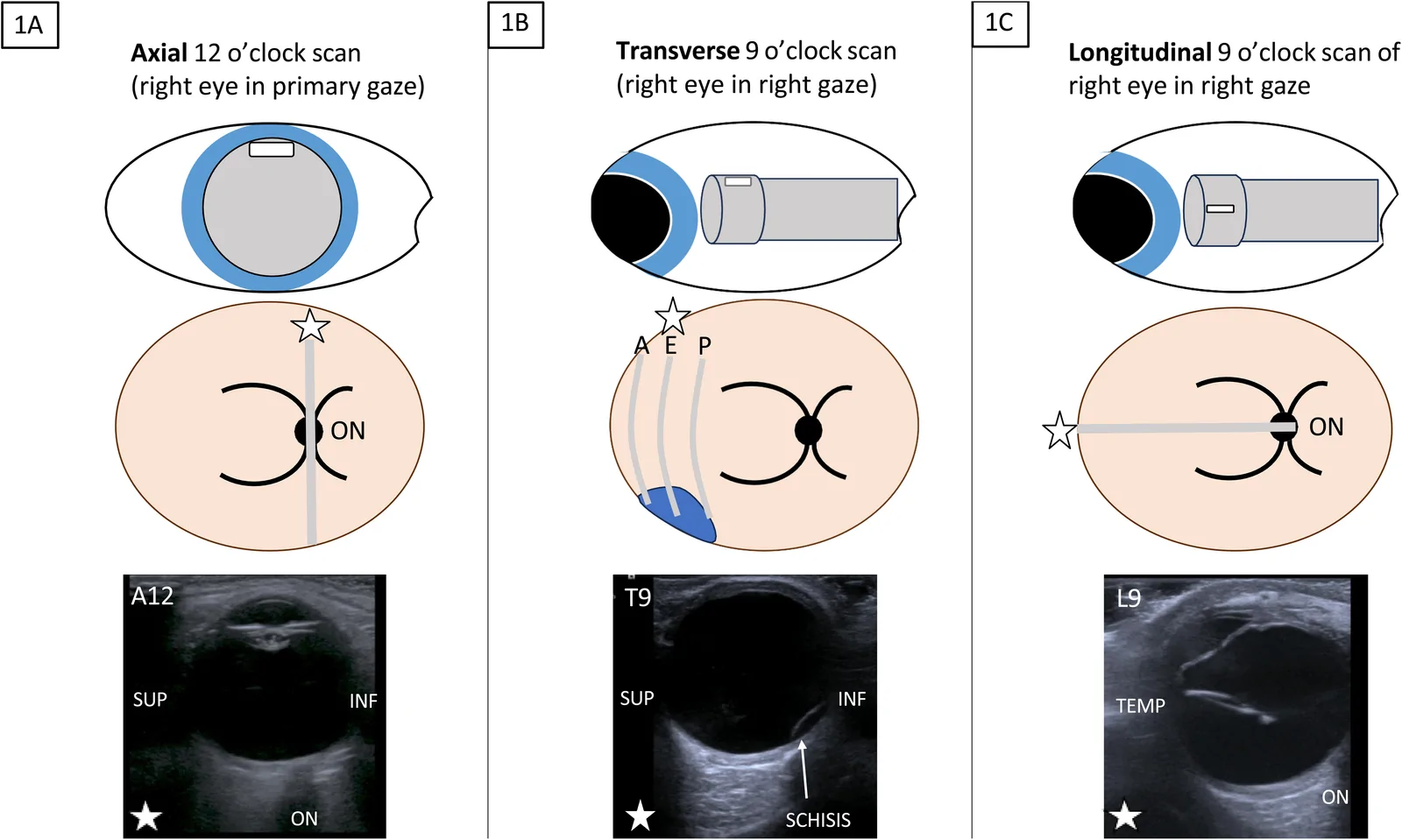
B-scan examination in different orientations produce different views (axial, transverse or longitudinal) of the globe. (A) Vertical axial scan is performed by placing the ultrasound probe in the centre of the cornea, with the marker (white rectangle) pointing superiorly at 12 o‘clock. The area examined is illustrated by the gray line on the fundus. The image orientation is indicated by the star on the fundus image and the left side of B-scan image. (B) Transverse 9 o’clock scan is performed by placing the ultrasound probe at 3 o’clock position, with the marker pointing superiorly. (C) Longitudinal 9 o’clock scan is performed by placing the ultrasound probe at 3 o’clock position, with the marker pointing at the limbus.

Ultrasound of the eye is performed using sterile gel. We recommend starting with a horizontal axial scan with the eye in the primary position to check for pathologies affecting the macula and optic nerve before assessing the eye in at least four positions of gaze using the probe in the transverse orientation. When performing axial and transverse scans, the probe is conventionally used with the marker pointing superiorly or nasally but there is variation in practice (Figure 1). The placement of the ultrasound probe marker determines the position of the macula on the horizontal axial scan. When performing horizontal axial scan on the left eye with the marker pointing towards 9 o’clock, the nasal retina is visualised on the left (or upper) part of the B-scan image followed by the optic nerve, macula and finally the temporal peripheral retina on the right (or bottom) part of the image.

The longitudinal scan is then carried out with the probe marker at the limbus to examine the vitreoretinal structure at each clock-hour meridian; this is useful for very anterior pathologies. On the longitudinal scan, the peripheral retina is visualised on the left (or upper) part of the B-scan image whereas the posterior pole and optic nerve are visualised on the right (or lower) part of the image (Figure 1C). A particularly useful scan to assess the macula status is the longitudinal macula scan (LMAC), performed with the patient's eye looking temporally and the probe at nasal limbus with the marker pointing towards the limbus i.e. temporally (Figure 1C). LMAC scan avoids scanning through the lens and provides a clear image of the macula and posterior pole. On the LMAC scan, the macula is adjacent to the optic disc at the right (or bottom) part of the image, whilst the insertion of lateral rectus is visualised on the left (or upper) part of the image (Figure 1C). In retinal detachment assessment, LMAC scan can help to determine if macula is on or off. Dynamic b-scan imaging is crucial for assessing vitreous mobility and traction.

Ultrasonographic assessment of the posterior segment frequently uses 10–20 MHz sound waves. Higher-frequency sound waves penetrate less deeply and are reserved for the anterior segment i.e. ultrasound biomicroscopy (UBM). In VR conditions, UBM is useful for assessing lens position and for any anterior segment pathologies that may have posterior segment consequences such as cyclodialysis cleft.

## B-scan in fundus obscuring vitreous haemorrhage (FOVH)

In most individuals, PVD is of no serious consequence. However, in 7–10% of symptomatic individuals with presumed pathologically strong vitreoretinal attachment, PVD may cause retinal tears or detachment.^4,5^ The risk of retinal tears increases drastically to almost 50% in those presenting with vitreous haemorrhage (haemorrhagic PVD due to avulsion of retinal vessels).^5^ Therefore, in acute and unexplained fundus-obscuring vitreous haemorrhage (FOVH), B-scan is arguably the most essential imaging modality, as it helps to distinguish a PVD-related FOVH from a vitreous haemorrhage that is not associated with vitreous separation and traction.

## Vitreous haemorrhage in the presence of PVD

PVD is confirmed by visualising the thin and uniformly echogenic posterior hyaloid membrane, which is attached anteriorly at the vitreous base and is separated from the retina posteriorly by the (normally hypoechogenic) subhyaloid space (Figure 2A). The vitreous body is also normally hypoechogenic but may show diffuse mobile hyperechogenic opacities in the presence of intragel vitreous haemorrhage (VH). On dynamic scanning, PVD is highly mobile with ‘after-movements’ but the vitreous may remain adherent at pathological areas of vitreoretinal traction such as the anterior flap of a retinal tear (Figure 2B).

**Figure 2.**
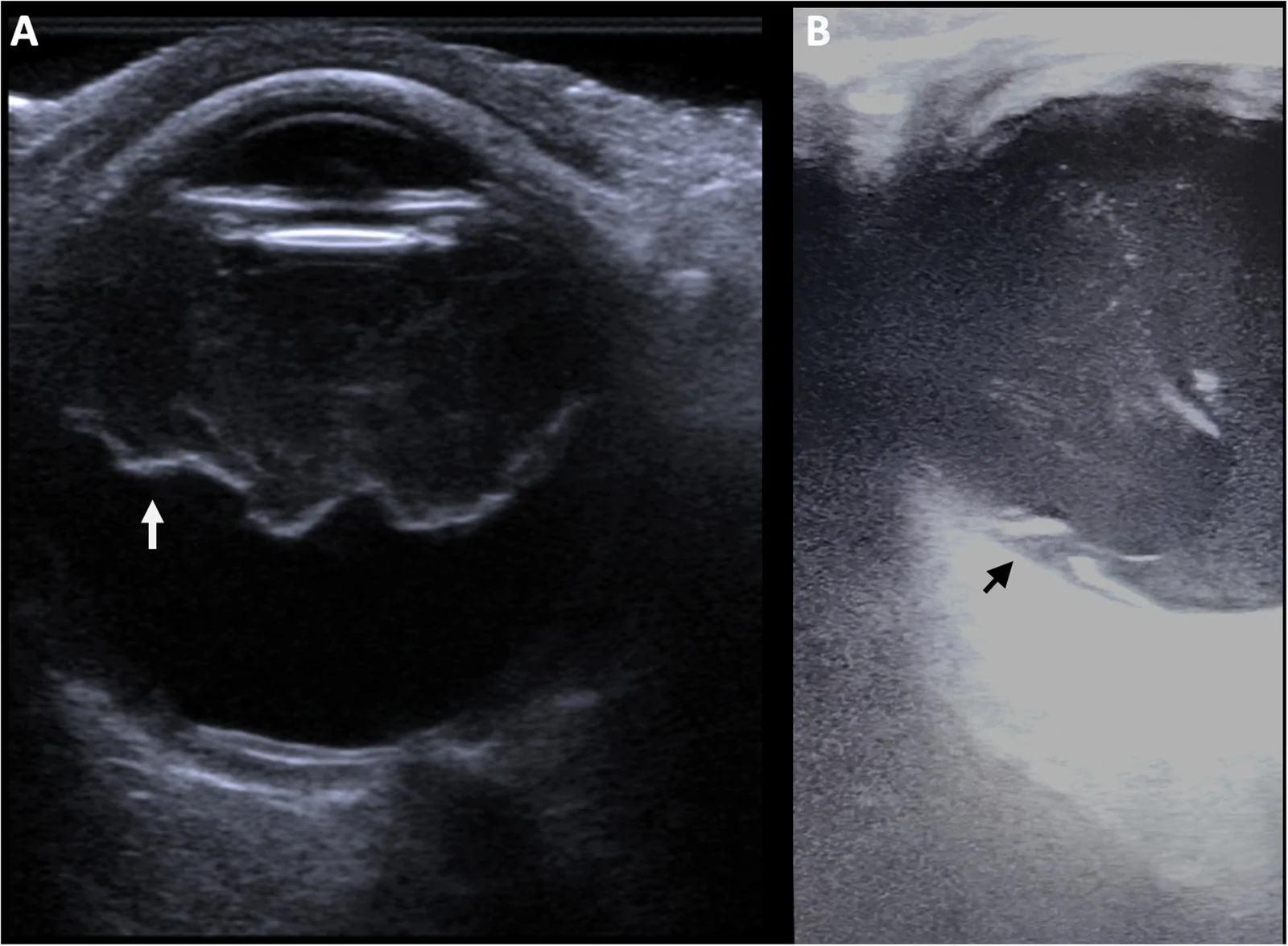
Fundus-obscuring vitreous haemorrhage. (A) Posterior vitreous detachment (white arrow) with intragel vitreous haemorrhage. (B) Haemorrhagic posterior vitreous detachment with localised vitreoretinal traction (black arrow) at the area of retinal tear and shallow subretinal fluid. The gain has been increased in Figure 2B to highlight the vitreous opacities (vitreous haemorrhage) at the expense of increased ‘noise’.

It is paramount not to mistake the posterior hyaloid for the retina on B-scan as this would trigger unnecessary vitrectomy. PVD demonstrates greater mobility and after-movements without any attachment to the optic disc. In contrast, rhegmatogenous retinal detachment (RRD) usually originates anteriorly from the site of retinal breaks, but its extent is limited posteriorly at the optic disc, even in total retinal detachment (Figure 3A and 4).

**Figure 3.**
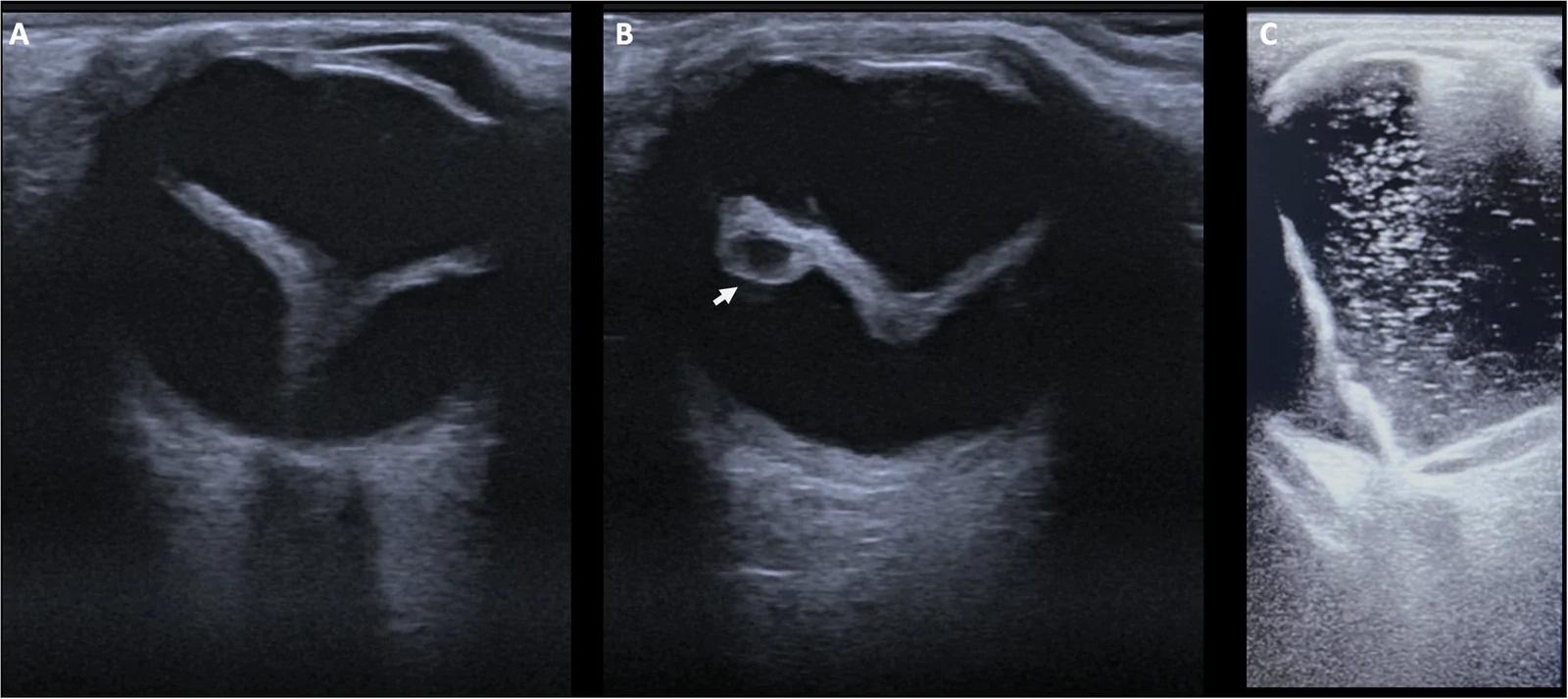
Features of severe or chronic retinal detachment. (A-B) B-scan showing total closed-funnel retinal detachment and a retinal cyst (white arrow), indicative of long-standing detachment. (C) B-scan showing poorly mobile total retinal detachment due to proliferative vitreoretinopathy in a 10-year-old patient with stickler syndrome, decompensated cornea, and emulsified silicone oil from previous vitrectomy surgery.

**Figure 4.**
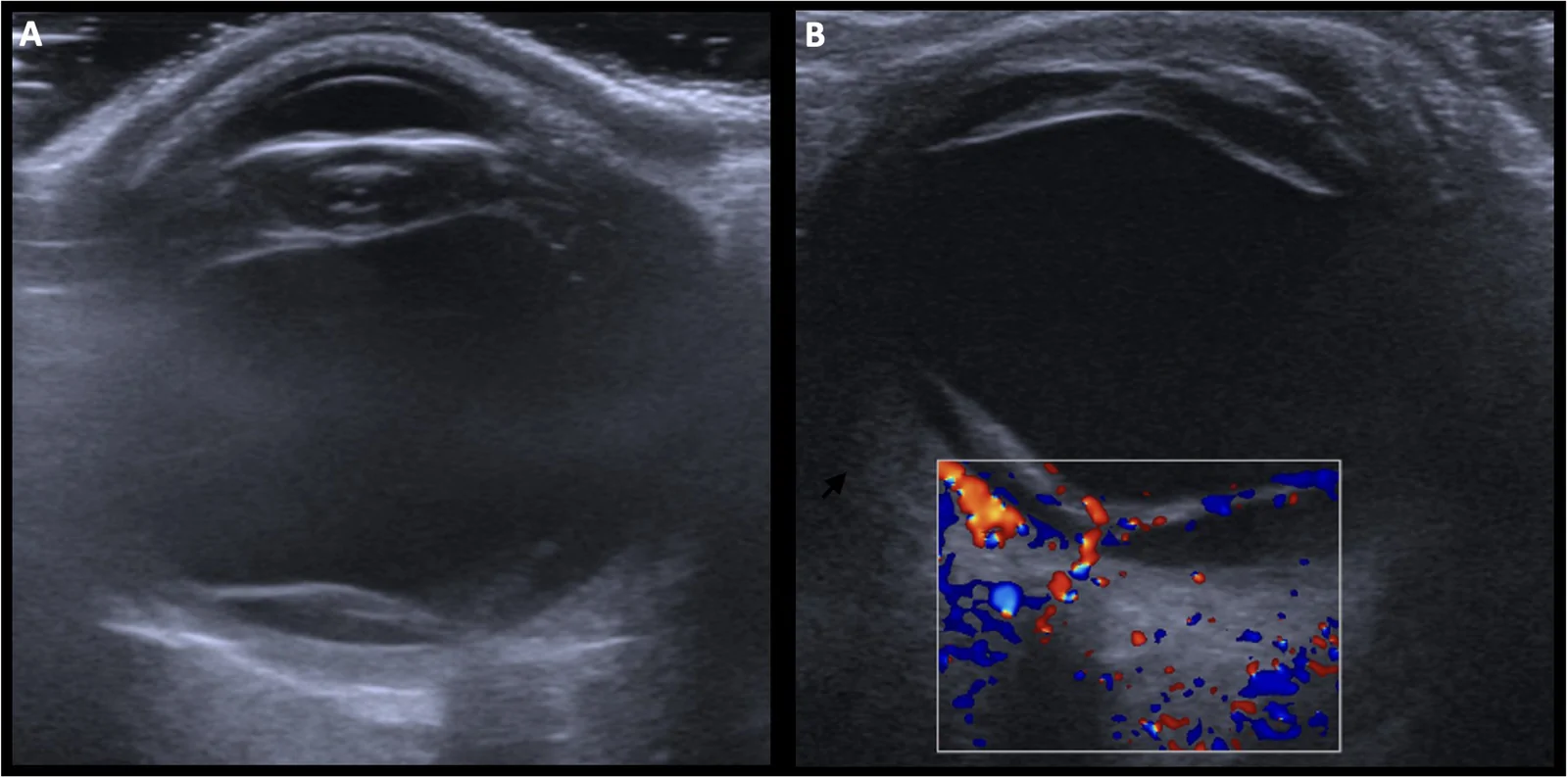
B-scan in an eye with macula-involving retinal detachment. (A) B-scan shows total RD with its posterior extent limited by the optic disc. (B) The use of doppler can conclusively demonstrate flow within detached retina, differentiating incomplete PVD from RD.

Since the PHM is less echogenic than the retina, it is helpful to start with high gain (e.g. 90 dB) to reveal PVD before reducing gain (e.g. 65 dB) to make the PHM (but not RD) undetectable.^6^ However, in long-standing vitreous haemorrhage or endophthalmitis, the PHM may be thickened and appear more hyperechogenic, especially inferiorly, mimicking RD (Figure 5). In such cases, colour doppler is useful for demonstrating flow in the retina but not in the avascular PHM (Figure 4B and 5A). Pulsed doppler ultrasound may further confirm retinal detachment by visualising a spectral waveform which synchronised with that of the central retinal artery.^7^ However, its use may be limited to skilled operators.

**Figure 5.**
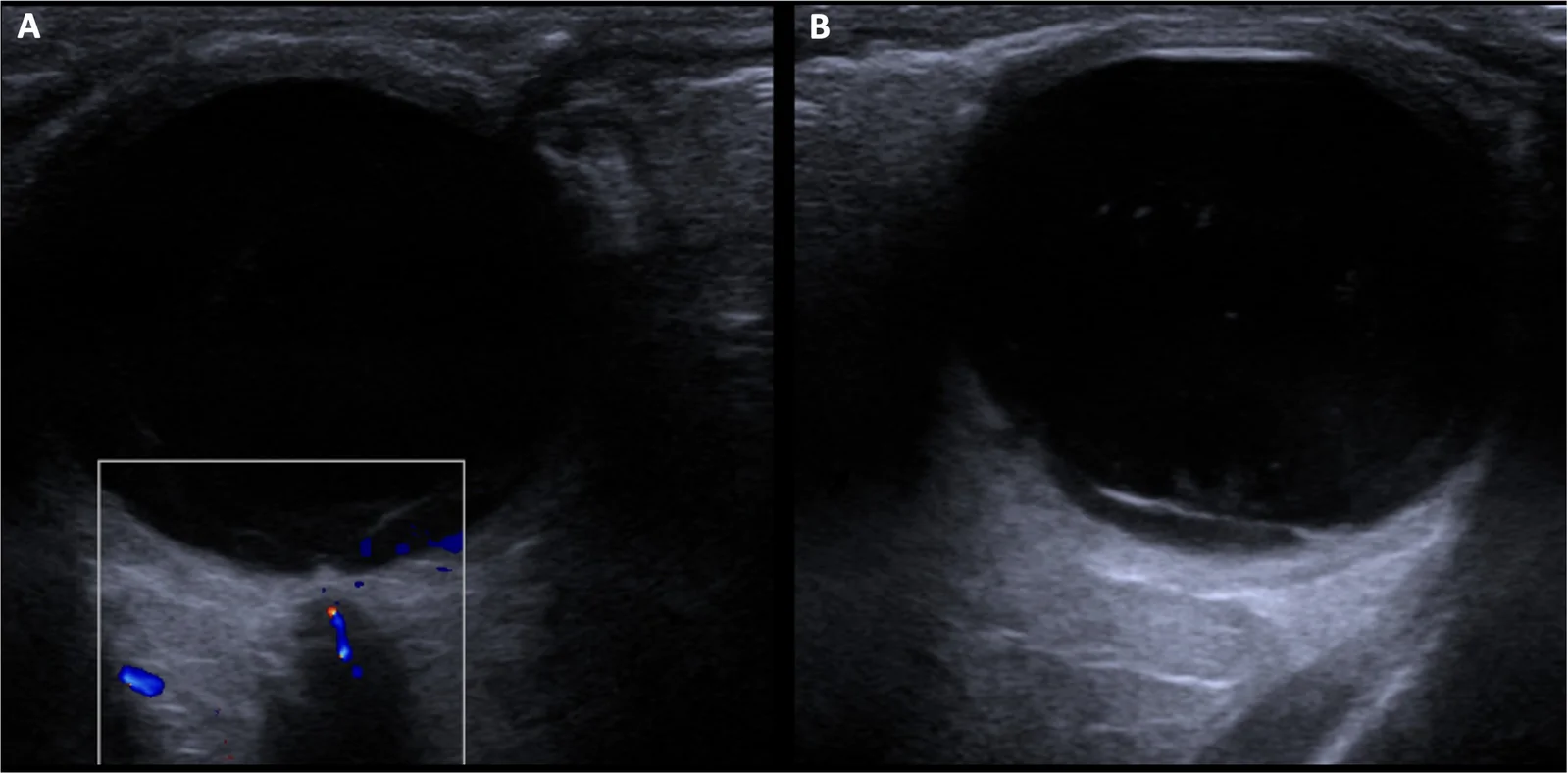
B-scan in an eye with long-standing vitreous haemorrhage secondary to disc neovascularisation without retinal detachment. (A) B-scan shows the posterior hyaloid is attached (incomplete PVD) to fibrovascular peg at the disc. The gain has been reduced such that posterior hyaloid is barely echogenic and vitreous haemorrhage not appreciable. The colour doppler does not show flow along the avascular posterior hyaloid, thus excluding RD. Blood flow is seen within the optic nerve only. (B) There is thickened hyaloid membrane inferiorly in the same eye due to long-standing vitreous haemorrhage.

Although the presence of retinal tears or RRD on B-scan should prompt early vitrectomy in FOVH, it is important to acknowledge that B-scan may miss small tears. In an undifferentiated cohort of 144 eyes undergoing vitrectomy for FOVH, preoperative B-scan showed a poor sensitivity of 58% and a specificity of 88% in identifying retinal tears or RD.^8^ Another study by Tan et al. in the University of Amsterdam also found low sensitivity (55.9%) of ultrasound in detecting retinal tear in vitreous haemorrhage.^9^ Hence, acute and unexplained FOVH due to PVD may warrant urgent vitrectomy even if B-scan fails to identify any tears or RD.

## Vitreous haemorrhage in the absence of PVD

FOVH without PVD is unlikely to have tractional retinal tears (such as horseshoe tears) and vitrectomy to examine the underlying retina is usually not immediately required. The clinical history and contralateral eyes may provide clues to the underlying cause. For instance, Terson syndrome may be suspected in those with subarachnoid haemorrhage. In some cases, B-scan may reveal the underlying source of non-PVD-induced FOVH such as retinal neovascularisation or breakthrough VH from a subretinal haemorrhage.

B-scan in those with retinal neovascularisation from diabetes, vein occlusion, sickle cell retinopathy or other causes of retinal ischaemia may demonstrate vitreous attachment to the fibrovascular pegs (Figure 5). Doppler may potentially confirm vascular flow within the neovascular complex growing into the vitreous. In the absence of a good fundus view, B-scan is crucial to identify any coexisting tractional retinal detachment (TRD), which dramatically changes the surgical approach and prognosis. TRD typically appears as taut and immobile retinal elevation without corrugations (Figure 6B). The skill and experience of the operator can greatly influence the accuracy of B-scan in detecting TRD in dense VH.^10^ In one study of 88 eyes with diabetic VH who underwent vitrectomy, preoperative B-scan could identify coexisting TRD with a high specificity of 96.6% but only 72.4% sensitivity.^11^

**Figure 6.**
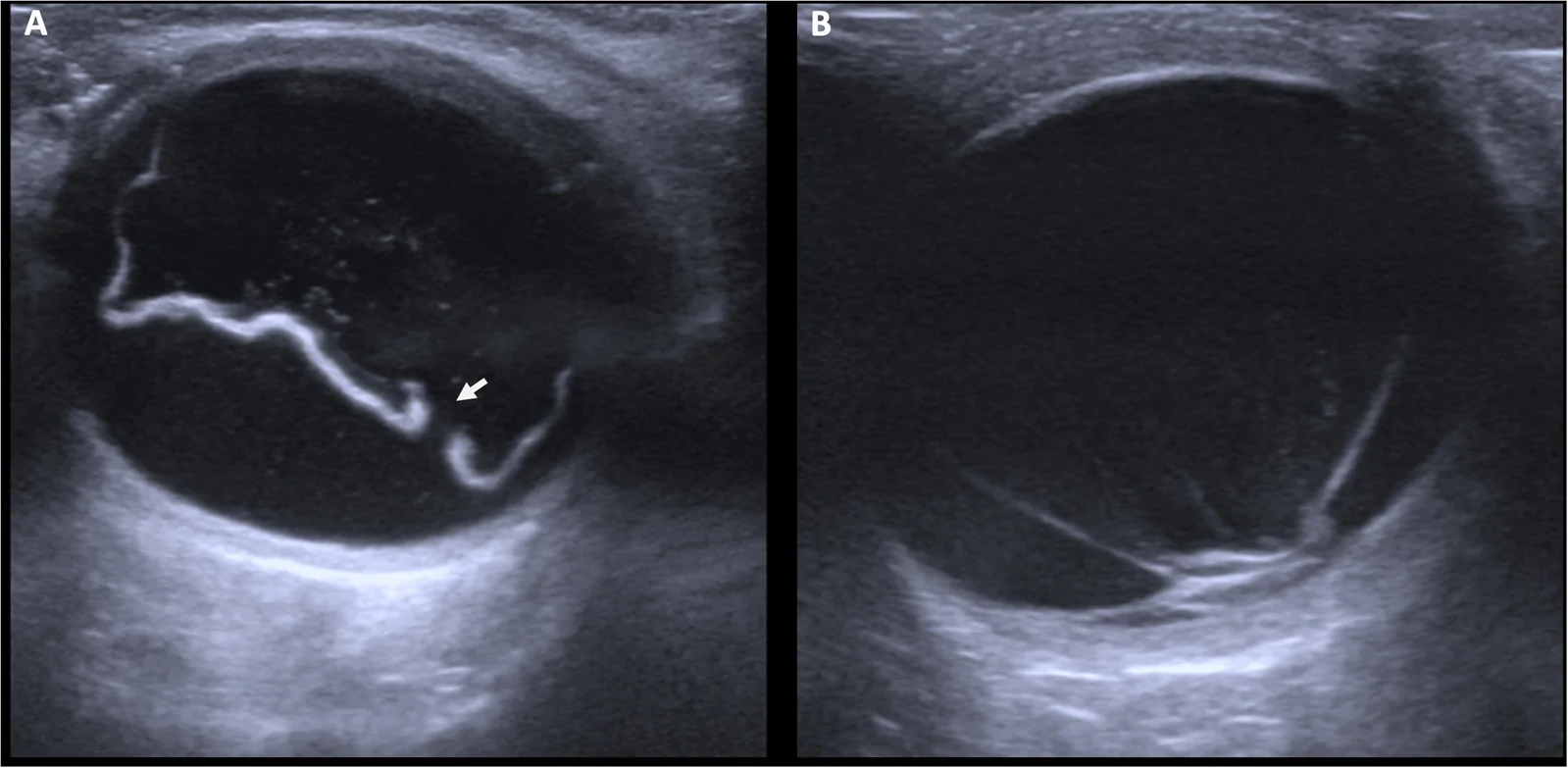
Rhegmatogenous and tractional retinal detachment. (A) B-scan shows bullous and corrugated appearance of rhegmatogenous RD, which was incidentally found in a patient with brunescent cataract. This transverse 12 o’clock scan shows a causative superotemporal retinal break within the detached retina (white arrow). (B) B-scan in a diabetic patient with vitreous haemorrhage and ‘table-top’ traction RD, showing taut and shallow retinal elevation.

Another non-PVD-induced mechanism of VH is breakthrough VH originating from subretinal haemorrhages in neovascular age-related macular degeneration, polypoidal choroidal vasculopathy, retinal artery macroaneurysm (Figure 7) and peripheral exudative haemorrhagic chorioretinopathy. B-scan can confirm the presence and location of subretinal haemorrhage. However, the goal of a B-scan is to determine if the macula is affected (i.e. submacular haemorrhage) as this would usually necessitate an urgent intervention.

**Figure 7.**
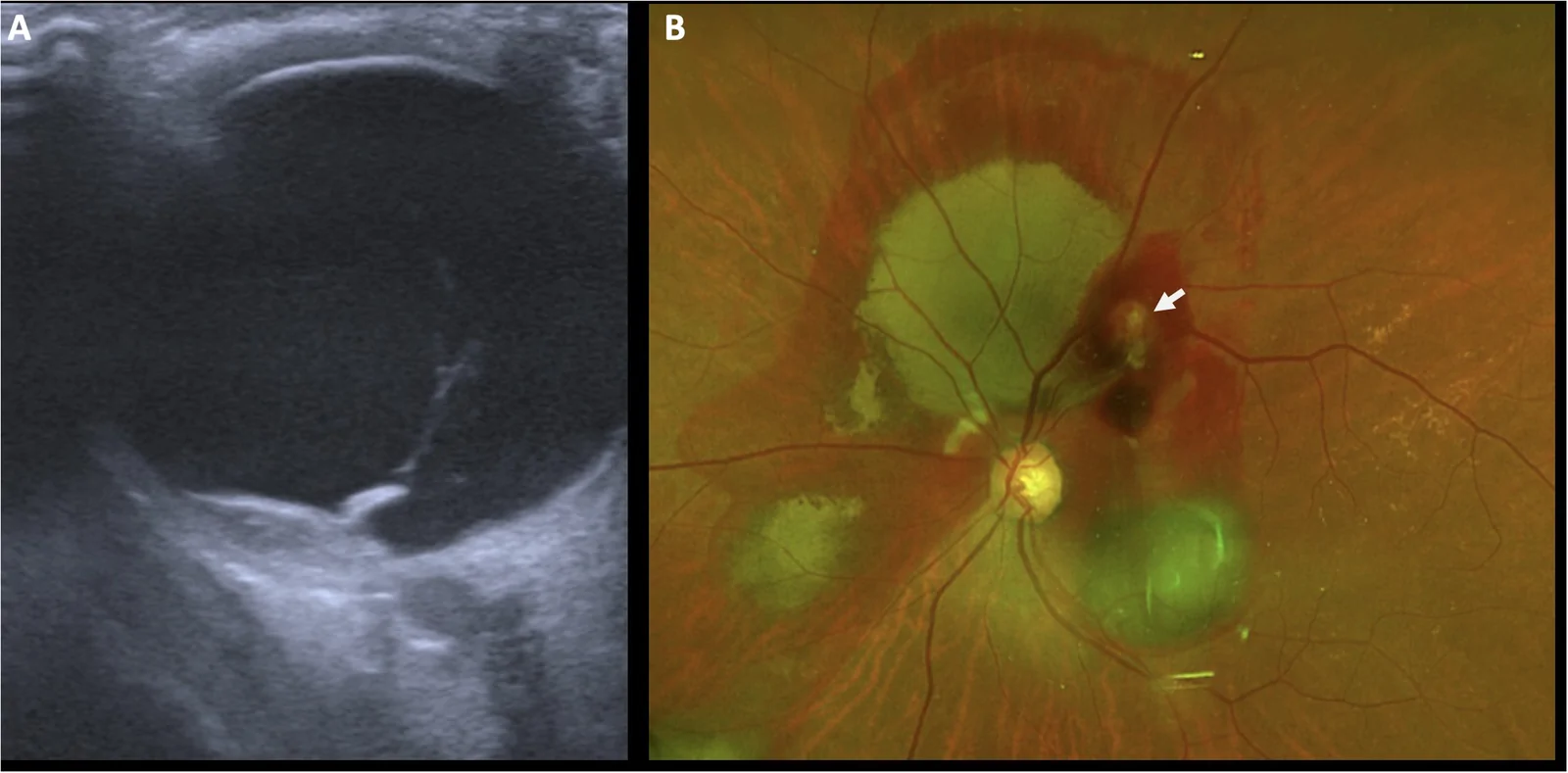
Breakthrough vitreous haemorrhage. (A) B-scan showing subretinal haemorrhage, pre-retinal clot (mimicking a flap tear) and breakthrough vitreous haemorrhage. (B) Ultrawide-field pseudocolour image of the same eye 4 weeks after pars plana vitrectomy, showing a multi-layered haemorrhage caused by a retinal artery macroaneurysm (white arrow).

## Choroidal detachment after anterior segment surgery

Choroidal detachment can develop following ocular surgery and may be easily mistaken for RD. Differentiating between the two can be challenging on fundus examination, particularly in the early post-operative period following anterior segment surgery, when media clarity is reduced. In such cases, B-scan can play a crucial role to make the correct diagnosis.

Unlike RRD, choroidal detachments appear thicker, non-mobile and smooth without corrugations. Classically, large choroidal detachments appear dome-shaped with multiple lobes seen in up to 4 quadrants. The posterior extent is usually limited to the mid-periphery where the choroid adheres most firmly to the underlying sclera at the ampullae of vortex veins. However, in severe cases, it may involve the posterior pole. Milder choroidal detachments may be subtle, shallow and show concavity. The internal reflectivity within choroidal detachment can be echolucent (choroidal effusion) or echogenic (blood, i.e. suprachoroidal haemorrhage) (Figure 8). B-scan is valuable not only for diagnosing choroidal detachment but also for monitoring the height and extent over time.

**Figure 8.**
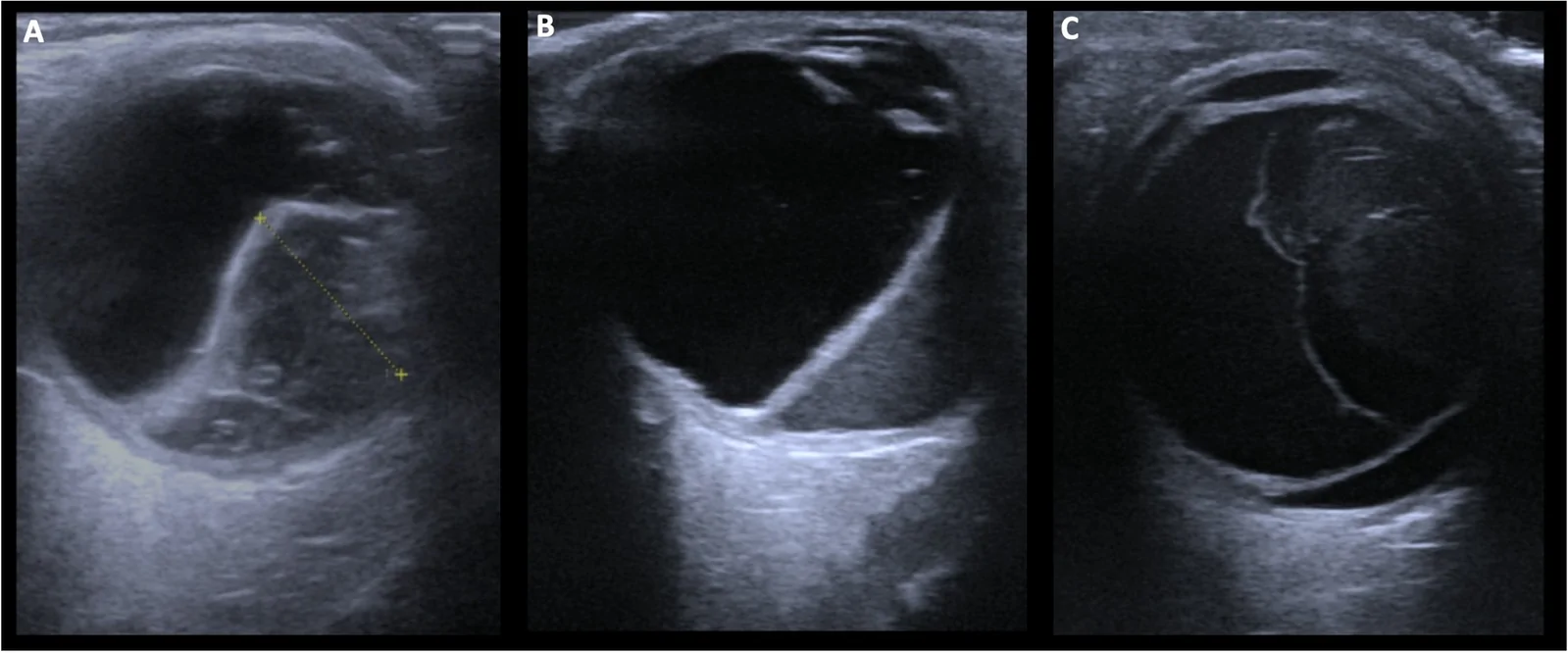
Choroidal detachment (suprachoroidal haemorrhage and choroidal effusion). (A) B-scan showing suprachoroidal haemorrhage with high and irregular internal reflectivity in early postoperative period and (B) subsequently, slight resolution was noted with reduction in height and more homogenous internal reflectivity. (C) B-scan in an eye with zone 2 globe rupture showing intra-gel vitreous haemorrhage and choroidal effusion (choroidal detachment with echolucent internal reflectivity).

In suprachoroidal haemorrhage, surgical intervention is typically considered with central retinal apposition (‘kissing choroidals’) or macula involvement, although differing opinion exist.^12^ Other considerations include flat anterior chamber with cornea-lens apposition, concomitant rhegmatogenous retinal detachment, breakthrough VH, vitreous or retinal incarceration in surgical wound, raised intraocular pressure or intractable pain despite medical therapy.

Serial B-scans are essential to determine the optimal timing to drain suprachoroidal haemorrhage, as waiting for clot liquefaction (usually 7–14 days) allows maximal drainage without excessive manipulation.^13^ In the early phase, blood clot within a suprachoroidal haemorrhage (SCH) appears ‘mass-like’ with high and irregular internal reflectivity. Over time, with spontaneous clot lysis, the internal reflectivity becomes less echogenic and more homogenous, eventually appearing as diffuse, low-reflective, mobile opacities.^12^ By identifying the deepest area of suprachoroidal haemorrhage, B-scan can also help guide the site of drainage sclerotomy.

It is worth noting that retinal and choroidal detachment are not mutually exclusive (Figure 9). Choroidal detachment can complicate retinal detachment and vice versa. Retinal detachment overlying suprachoroidal haemorrhage may be exudative or rhegmatogenous. Exudative retinal detachment is usually smooth, shallow and low-lying over areas of choroidal detachment, whereas RRD is more elevated, shows corrugations and may not be overlying the area of choroidal detachment. Exudative retinal detachment may regress as choroidal detachment improves, thus making this distinction is clinically important.^12^

**Figure 9.**
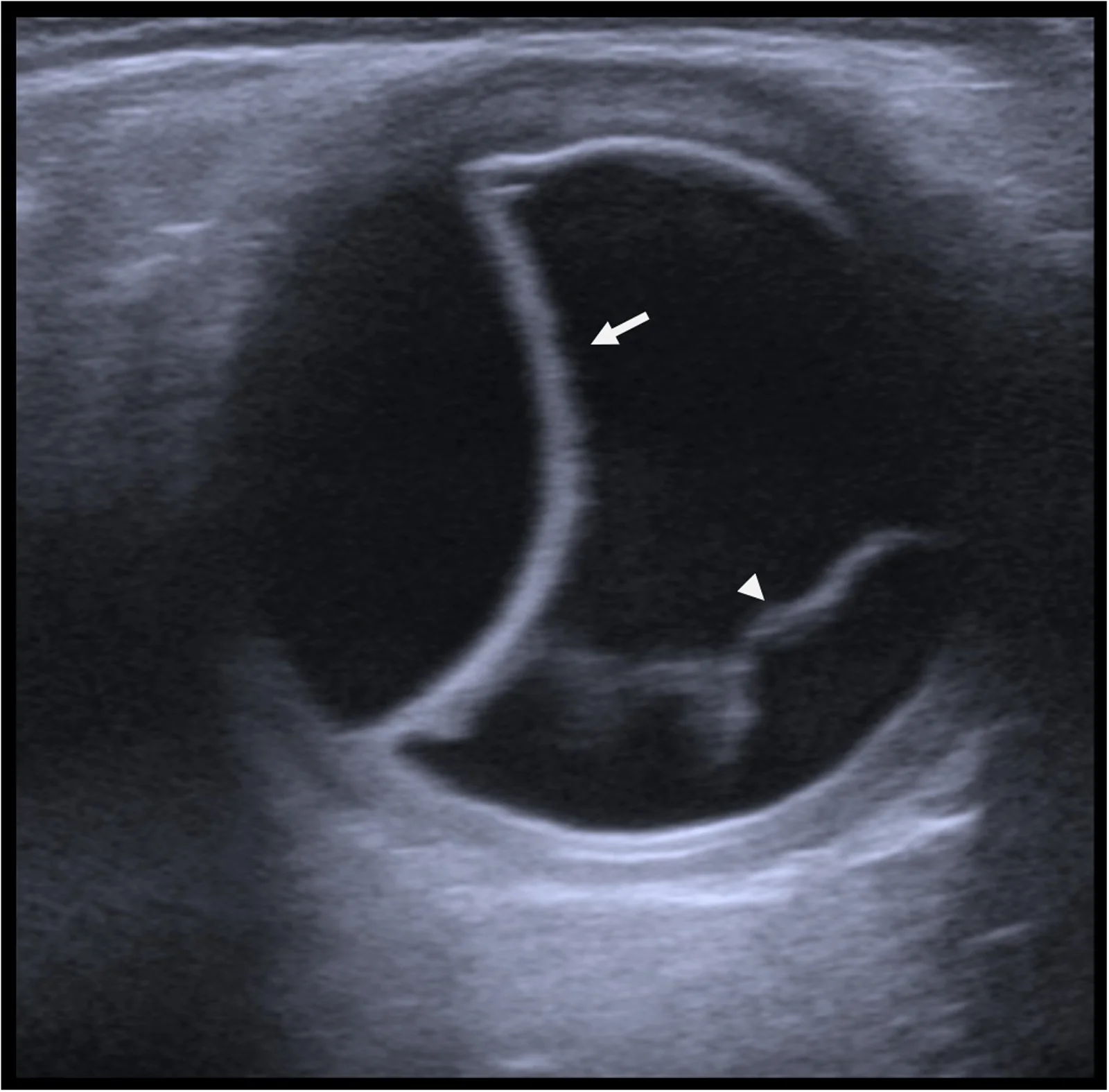
Concomitant choroidal detachment and retinal detachment. B-scan showing large choroidal effusion (white arrow) and concomitant exudative retinal detachment (arrowhead) in an eye post glaucoma drainage tube surgery. Fundus examination was possible in this patient, confirming the absence of retinal tear.

## UBM in hypotony assessment

The cause of choroidal effusion is often obvious, such as low IOP after glaucoma surgery. In choroidal effusion with no obvious cause found on history or slit-lamp examination, UBM may identify the reversible cause of hypotony such as cyclodialysis cleft.

UBM is also increasingly used to evaluate chronic hypotony in patients with uveitis. Common UBM findings in chronic uveitic hypotony include supraciliary effusion, epiciliary membrane with or without ciliary body traction, and ciliary body atrophy (blunting or reduction in the number or size of ciliary body indicating shutdown).^14^ It is thought that uveitic hypotony with only supraciliary effusion can be managed with medical therapy alone, but those with ciliary body traction from post-inflammatory epiciliary membrane require removal of tractional membranes.^14–16^ The role of UBM is to identify the underlying mechanisms of hypotony and in the latter, to identify the location and thickness of the tractional cyclitic membrane for surgical planning. Patients with concurrent ciliary body atrophy may also benefit from additional procedures such as silicone oil tamponade or encircling buckle.^14–17^ Intravitreal injection of viscoelastic sodium hyaluronate has also shown favourable results in several case series of eyes with chronic hypotony secondary to vitreoretinal surgery, uveitis or trauma.^17^

**RRD mimics:** exudative RD, schisis and pars plana cysts

Exudative retinal detachment is an important differential to rhegmatogenous retinal detachment. Clinical history and examination findings usually give clues to the underlying diagnosis. On B-scan, exudative RD appears as smooth convex, dome-shaped elevation that classically show gravitational change. Associated features such as vitritis, scleritis, and tumour are helpful clues towards exudative aetiology. Where a lesion is noted, doppler can identify internal vascularity, seen in vascular lesions or choroidal melanoma but not in haematoma (such as subretinal haemorrhage).

Retinoschisis appears thin, smooth (non-corrugated), and dome-shaped (Figure 10). Unlike RRD, retinoschisis shows minimal-to-no mobility or undulations on dynamic scan. B-scan can visualise that retinoschisis does not flatten with scleral indentation, whereas RRD does partially flatten. UBM may also provide further clues to differentiate retinoschisis from RD.^18^ For instance, in one study examining 25 eyes with retinoschisis and 23 eyes with RD, UBM found ‘retinal layers split’ in 72%, intraretinal pillars in 64%, and intraretinal cysts in 36% of eyes with retinoschisis. None of these findings were noted in eyes with RD.^18^

**Figure 10.**
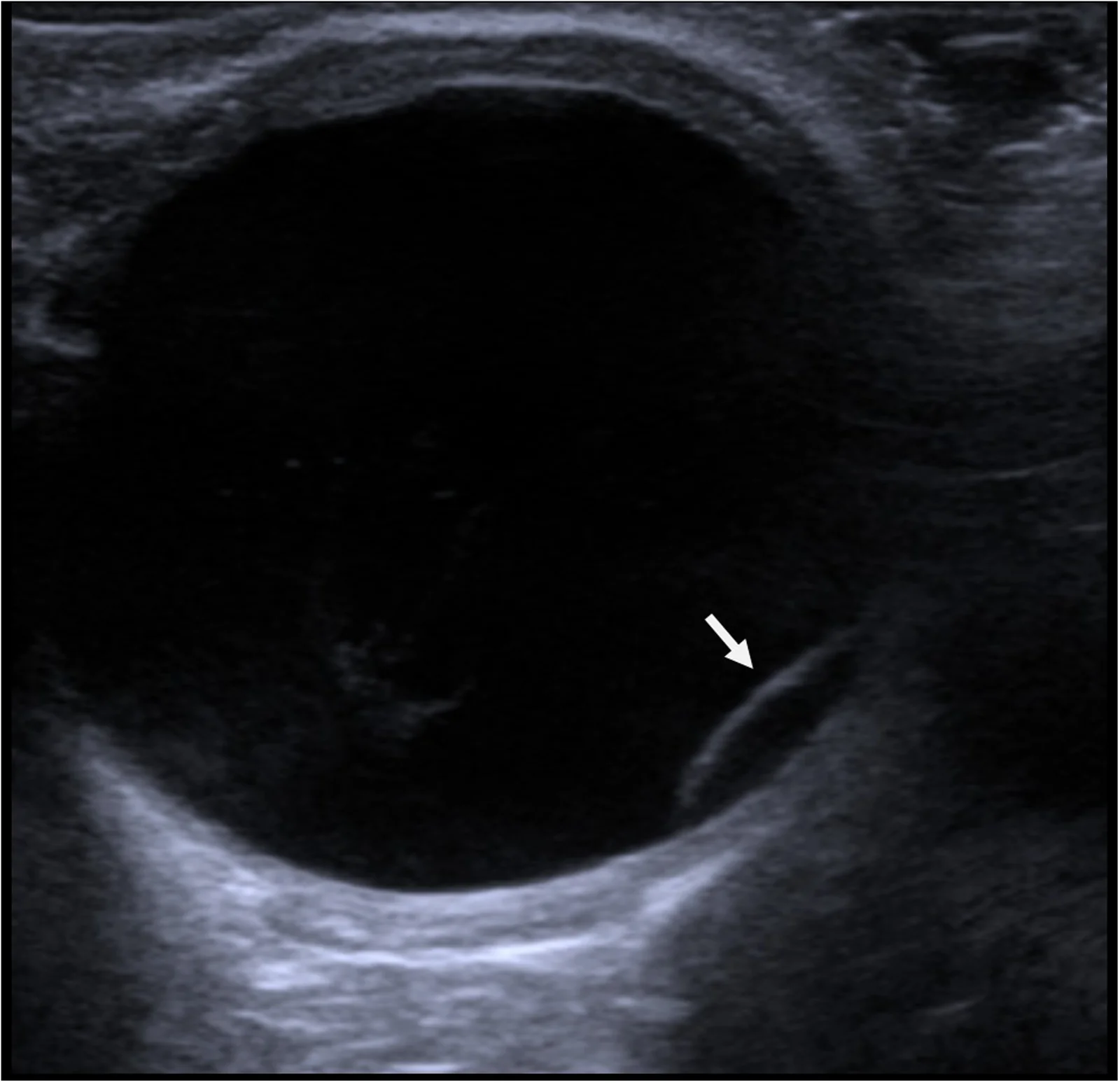
RD mimics. Transverse 5 o’clock B-scan of retinoschisis in the inferotemporal quadrant shows smooth thin echolucent elevation (white arrow).

Large pars plana cysts (or ‘giant’) may also mimic RD. B-scan and UBM can confirm that these cysts are within the pars plana rather than the retina.

## B-scan in the management of rhegmatogenous retinal detachment

B-scan can sometimes be used to guide the management of RRD beyond its initial diagnosis. Since scleral buckling is preferred to vitrectomy for RRD without PVD such as those caused by round retinal holes,^19,20^ the assessment of the PVD status is crucial. In equivocal cases, B-scan may therefore be needed even in cases with a good view of the fundus.

In the absence of PVD, RRD usually results from round atrophic holes or dialysis. While atrophic holes are usually too small to be visualised on B-scan, retinal dialysis can be appreciated as circumferential retinal elevation at the ora serrata, with the vitreous base attached to its posterior flap (Figure 11). UBM can also visualise retinal dialysis as a retinal separation from ora serrata (Figure 11). UBM has also been used in detecting very anteriorly located tears within the non-pigmented epithelium of pars plana, confirming the RRD diagnosis (excluding exudative RD) and guiding scleral buckling site in a patient with RD and schwatz-matsuo syndrome.^21^

**Figure 11.**
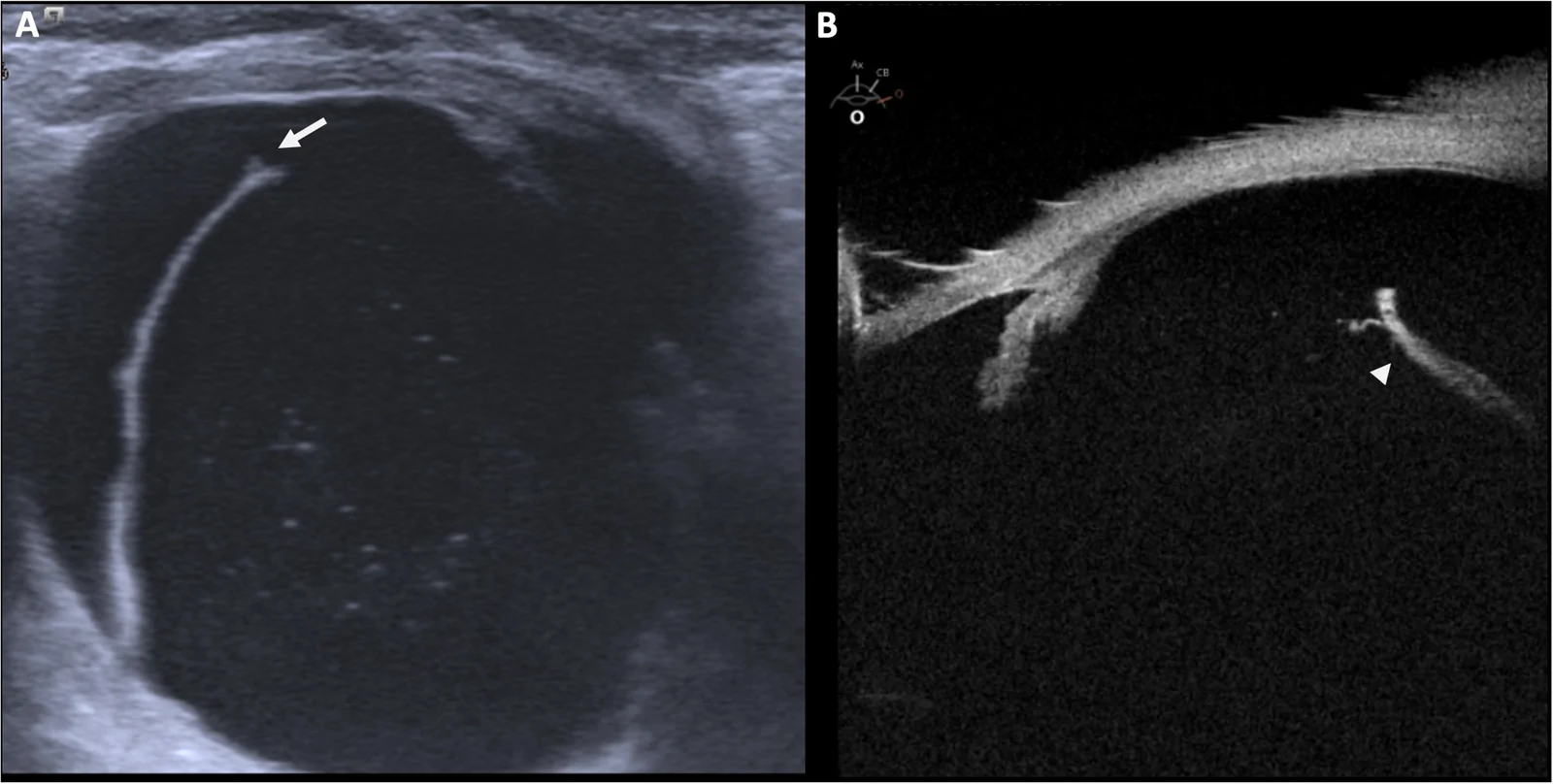
Detachment from retinal dialysis. (A) B-scan shows retinal dialysis from ora serrata (white arrow) and the extent of retinal detachment posteriorly. There is no PVD visible. (B) UBM can be used to visualise the retinal dialysis (white arrowhead) from ora serrata.

B-scan may also provide prognostic information by identifying features of chronicity such as poor mobility of the retina due to proliferative vitreoretinopathy (Figure 3), retinal cysts and open-funnel RRD.^22^ This helps to inform preoperative discussion especially when consenting patients ‘incidentally’ found to have RRD as part of the assessment of hypermature cataract. Such information can guide treatment decisions and triaging in a resource-limited setting. The finding of complex closed-funnel RRD may suggest ‘inoperability’ due to very low chance of success.

## Vitritis and endophthalmitis

Exogenous post-surgical endophthalmitis frequently presents with poor fundal view due to hazy cornea and intraocular inflammation. B-scan can improve the certainty of diagnosis by demonstrating vitreous involvement in suspected endophthalmitis as inflammatory vitreous debris (vitritis) are hyperechoic and can be loculated (36%) or membranous (70%)^23^ (Figure 12A-B). Conventionally, B-scan is recommended before and after intravitreal injection of antibiotics or surgical management of endophthalmitis because RD complicates 8% of endophthalmitis cases and confers poorer prognosis.^24^ Other B-scan findings in endophthalmitis include choroidal detachment (as high as 14.8% in one study), choroidal thickening (16.7%) and thickened posterior sclera with fluid in sub-tenon's space (13%).^23^ Preoperative B-scan is particularly useful for guiding the choice of the sclerotomy sites in endophthalmitis complicated with retinal or choroidal detachment.^25^ It is crucial that clinicians adequately disinfect probe following the ultrasound of eyes with endophthalmitis.

**Figure 12.**
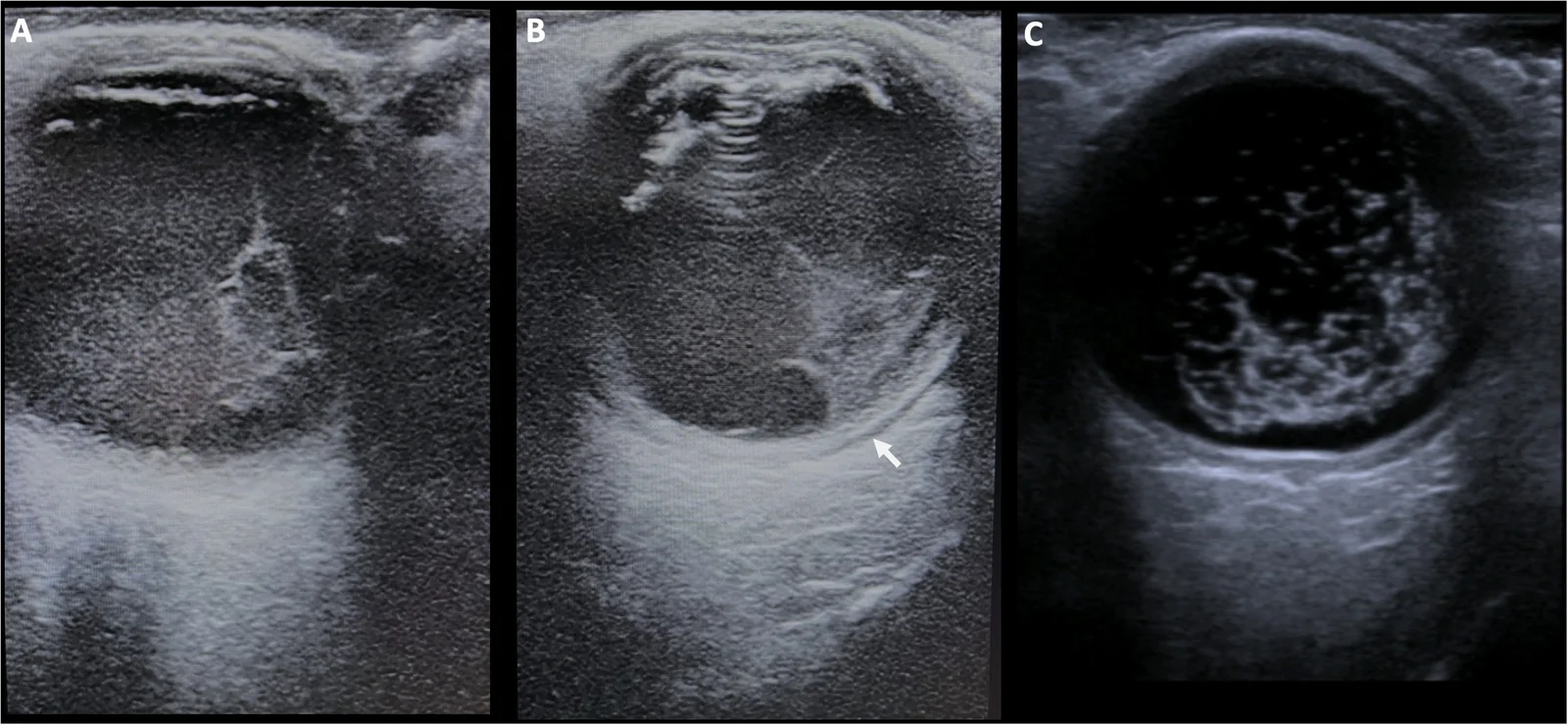
Vitreous changes. (A-B) B-scan in a patient with endophthalmitis showing marked membranous vitreous opacities that were loculated, choroidal thickening and fluid in subtenon's space (white arrow). (C) B-scan in a patient with hypopyon and asteroid hyalosis with discrete hyperechoic opacities and a clear echolucent zone between vitreous bodies and the retina.

## Asteroid hyalosis

In the absence of a fundal view, asteroid hyalosis can be easily confused with vitritis (Figure 12C). In contrast to vitritis, asteroid hyalosis appears as multiple discrete and relatively homogenous hyperechogenic bodies within an otherwise hypoechoic vitreous cavity. A clear hypoechoic zone between asteroid hyalosis and the retina can be seen, especially in patients with PVD. On dynamic B-scan, the asteroid bodies are mobile, producing a comet-tail artefact and a unique sparkling appearance, akin to ‘particles in a snow globe’.^26^ The latter is not observed in vitritis or vitreous haemorrhage.

## Dropped nucleus and lens dislocation

Dropped nucleus or dislocated intraocular lens is usually recognised at the time of complicated cataract surgery; however, the fundal view can be limited in the early postoperative period due to corneal oedema (Figure 13). Hence, B-scan is mainly used to check for retinal tear or detachment that may result from vitreoretinal traction associated with any vitreous loss into the anterior segment.

**Figure 13.**
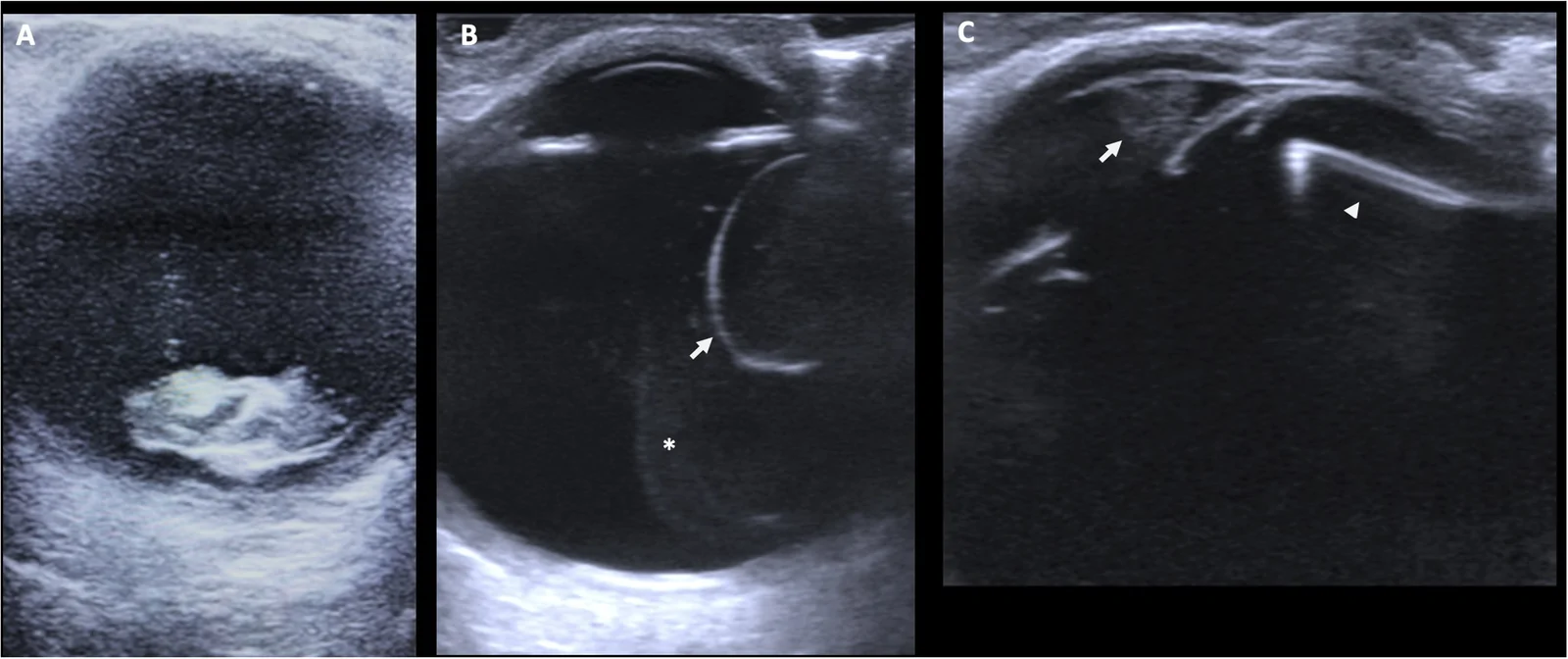
Subluxated or dislocated lens. (A) B-scan showing a dropped nucleus without intact capsule. (B) B-scan of an eye which suffered blunt trauma causing a dislocated lens with intact capsule (white arrow) and inferior vitreous haemorrhage (asterisk) (C). B-scan showing retained lens matter in the anterior chamber (white arrow) and an intraocular lens dislocated into the vitreous chamber (white arrowhead).

In cases of traumatic lens dislocation with associated vitreous haemorrhage, B-scan can evaluate the integrity of the lens capsule. Those with intact lens capsule require less urgent intervention than those with compromised lens capsule, as the former is less likely to trigger an inflammatory cascade and IOP rise (Figure 13).

In phacolytic glaucoma, where the aqueous becomes cloudy due to lens protein leakage, the integrity and stability of the hypermature lens can be assessed with B-scan which will also demonstrate any lens subluxation (Figure 14). In such cases, an alternative to standard phacoemulsification cataract surgery may be necessary in the presence of weak zonular support.

**Figure 14.**
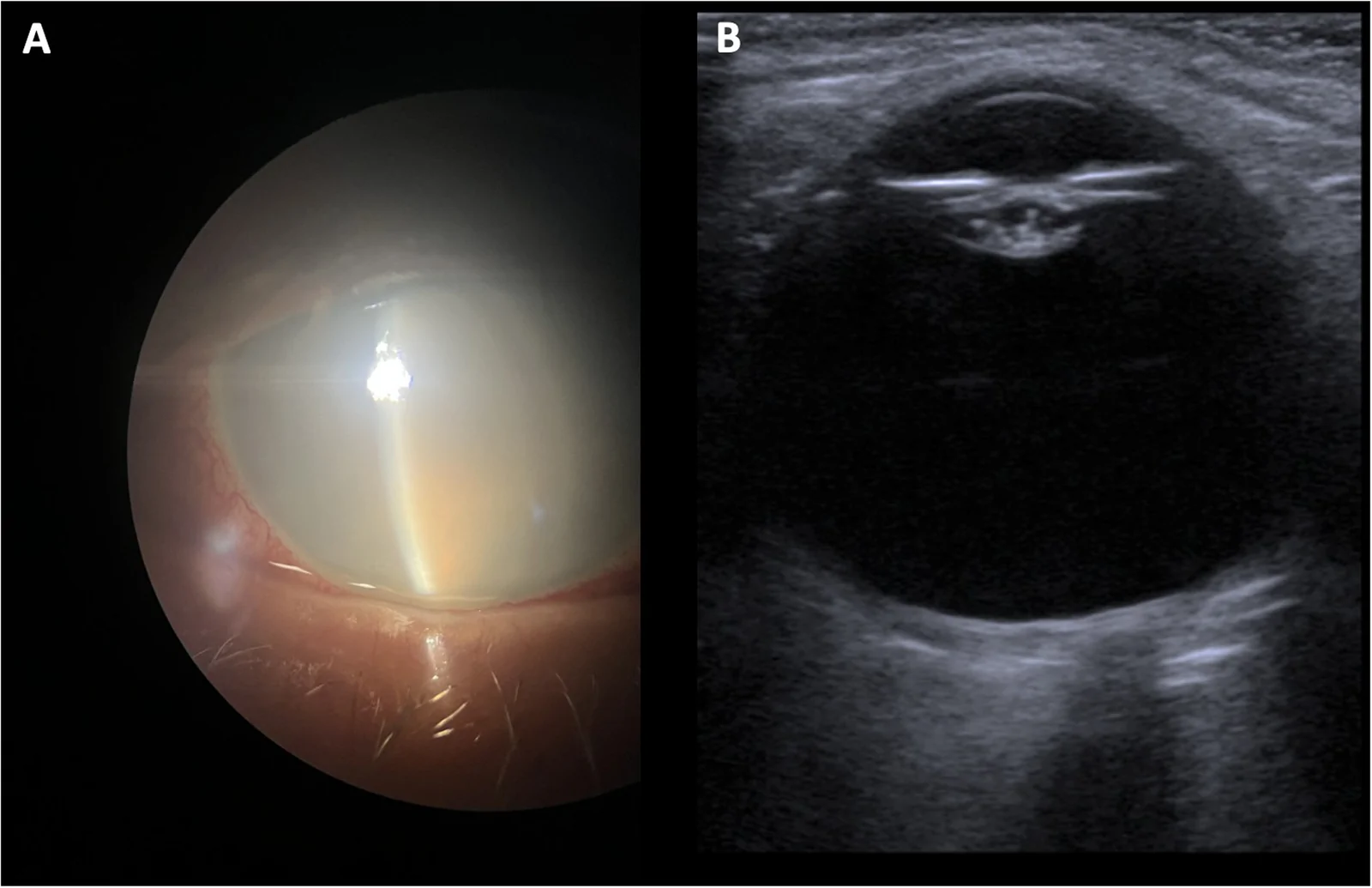
Hypermature cataract. (A) Anterior segment photograph of phacolytic glaucoma with milky aqueous appearance due to leakage of lens proteins from hypermature lens. (B) B-scan showing a hypermature cataract but no vitritis, ruling out endophthalmitis.

## Malpositioned intraocular lens

Malpositioned intraocular lens (IOL) may chafe uveal tissues, causing chronic inflammation and uveitis-glaucoma-hyphema (UGH) syndrome (Figure 15). Even in eyes without clinically evident iris transillumination defects on slit-lamp examination, UBM can directly visualise the abnormal mechanical contact between the intraocular lens and uveal tissues.^27^ In a case series of 20 patients with unexplained chronic inflammation, recurrent hyphaema, raised IOP, or recurrent vitreous haemorrhage following phacoemulsification, UBM confirmed malposition of lens haptics (anterior-chamber IOL, single-piece or three-piece IOL) within or against the iris (75%), ciliary body processes (35%) or pars plana (10%). The two commonest scenarios in this case series are 1) a single-piece IOL with one haptic misplaced in the sulcus and 2) a sulcus IOL with the haptic embedded or extending into the ciliary body processes. Of interest, in the context of undisplaced IOL, Soemmering's ring has been reported to contact posterior iris surface and induce UGH syndrome; this can be appreciated on UBM.^28^

**Figure 15.**
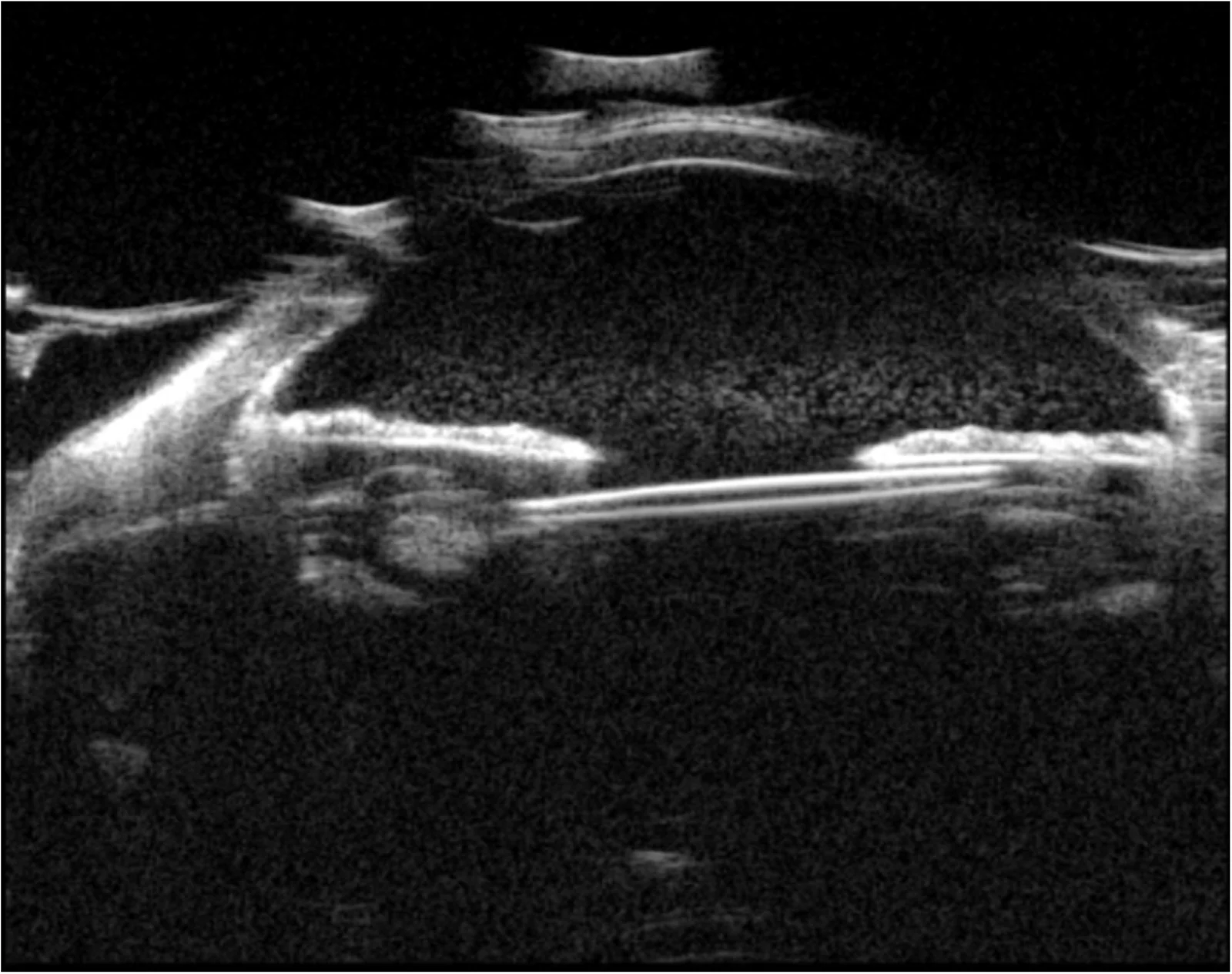
Malpositioned intraocular lens. This patient with a known history of glaucoma presented with microhyphema and vitreous haemorrhage. B-scan confirmed a single-piece intraocular lens with optic tilted anteriorly due to one haptic being misplaced in the sulcus. Microhyphema can be appreciated by opacities within the anterior chamber. This is a case of uveitis-glaucoma-hyphaema (UGH) plus syndrome.

UBM can also be used to check for horizontal or vertical tilt of scleral-fixated intraocular lens and its haptic position (within ciliary sulcus, posterior to ciliary body or through ciliary body).^29^ Notably, the subluxated lens may be more noticeable when the patient is lying flat (Figure 16).

**Figure 16.**
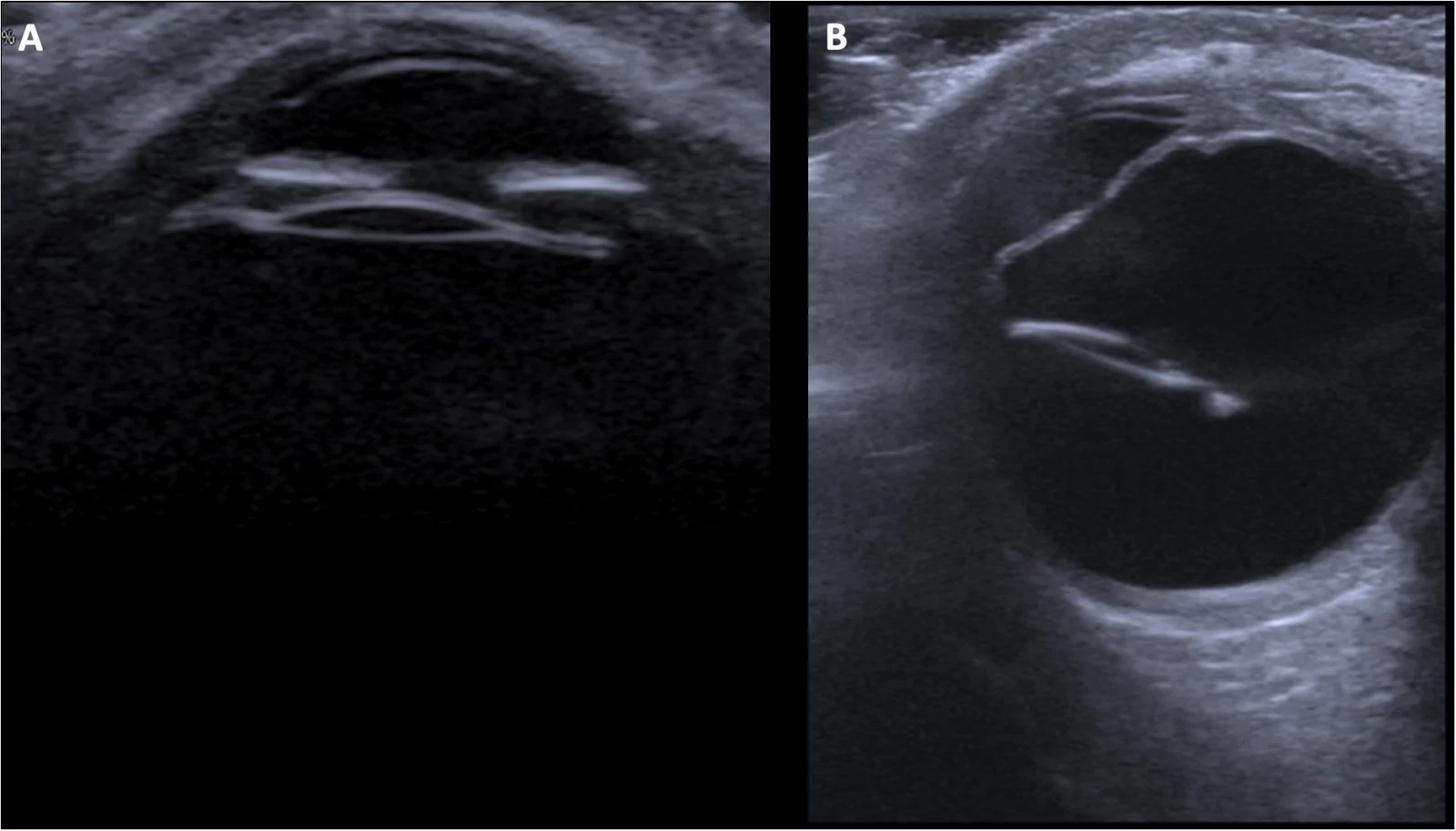
Subluxed intraocular lens. (A) B-scan in a patient with decentred and posteriorly tilted scleral-fixated intraocular lens. This was more pronounced in supine position than in sitting up. (B) The nasal T-haptic was not fixated onto the sclera.

## Conclusion

USG is quick and safe, with broad applications in the assessment and management of vitreoretinal conditions and complications of anterior segment surgery. However, the quality of imaging and interpretation is often operator-dependent. An improved understanding of the strengths and weaknesses of B-scan, doppler and UBM is valuable in complex cases with diagnostic uncertainty.

## Data Availability

Anonymised data presented in this study are available on reasonable request from corresponding author and approval by the relevant hospital institutions
